# Potassium channel-related genes are a novel prognostic signature for the tumor microenvironment of renal clear cell carcinoma

**DOI:** 10.3389/fonc.2022.1013324

**Published:** 2022-09-28

**Authors:** Rui Zeng, Yi Li, Dong-ming He, Meng-zhu Sun, Wen-qing Huang, Yu-hang Wang, Yu-min Zhuo, Jun-jiang Chen, Tai-heng Chen, Jing-hui Guo, Jun Huang

**Affiliations:** ^1^ Department of Physiology, School of Medicine, Jinan University, Guangzhou, China; ^2^ Department of Urology, The First Affiliated Hospital of Jinan University, Guangzhou, China; ^3^ Department of Transfusion Medicine, Shenzhen Hospital, Southern Medical University, Shenzhen, China

**Keywords:** clear cell renal cell carcinoma (ccRCC), prognostic signature, potassium channel, tumor microenvironment, immunotherapy

## Abstract

Clear cell renal cell carcinoma (ccRCC) accounts for 80% of renal cell carcinomas (RCCs), and its morbidity and prognosis are unfavorable. Surgical resection is the first-line treatment for ccRCC, but the oncogenesis of ccRCC is very complex. With the development of high-throughput sequencing technology, it is necessary to analyze the transcriptome to determine more effective treatment methods. The tumor microenvironment (TME) is composed of tumor cells, various immune-infiltrating cells, fibroblasts, many cytokines, and catalysts. It is a complex system with a dynamic balance that plays an essential role in tumor growth, invasion, and metastasis. Previous studies have confirmed that potassium channels can affect the immune system, especially T lymphocytes that require potassium channel activation. However, the effect of potassium channels on the TME of ccRCC remains to be studied. Therefore, this study aims to construct a prognostic signature for ccRCC patients based on potassium ion channel-related genes (PCRGs), assess patient risk scores, and divide patients into high- and low-risk groups based on the cutoff value. In addition, we investigated whether there were differences in immune cell infiltration, immune activator expression, somatic mutations, and chemotherapeutic responses between the high- and low-risk groups. Our results demonstrate that the PCRG signature can accurately assess patient prognosis and the tumor microenvironment and predict chemotherapeutic responses. In summary, the PCRG signature could serve as an auxiliary tool for the precision treatment of ccRCC.

## Introduction

Renal cell carcinoma (RCC) is the most common malignant tumor in the urinary system, and 80% of RCCs are the clear cell renal cell carcinoma (ccRCC) pathological type. This percentage is far more than the that for the mixed cell type, granulosa cell type, and undifferentiated cell type (1). According to the World Health Organization and the International Society of Urological Pathology (WHO/ISUP) classification system (2), ccRCC can be divided into four grades (grades I-IV). Even the first-line treatment of ccRCC is surgery (3), however, nearly one-third of patients with ccRCC already have metastasis at the first diagnosis, and the clinical curative effect is limited in patients with metastasis, even when combined with chemotherapy and immunotherapy. The first line of treatment for metastatic RCC patients is immune checkpoint inhibitors (ICIs) in combination with tyrosine kinase inhibitors (TKIs) (4); however, patients with locally advanced or metastatic RCC have a poor prognosis. Before metastasis, the overall survival rate for RCC is 74%, and for patients with metastasis, the 5-year survival rate decreases to 8% (5). Thus, it is important to identify new biomarkers or targets to increase the early diagnosis rate of ccRCC and enhance the effect of early intervention treatment.

Recently, the tumor microenvironment (TME), which includes tumor-infiltrating immune cells (TICs), has been shown to play a decisive role at all stages of tumor progression (6–8). ccRCC is a highly immune-infiltrated tumor, and the high immune infiltration of ccRCC has been proven in multiple studies (9). Immune cells play a key role in anticancer immunity. By immunomonitoring, TICs could predict the prognosis of ccRCC patients and enhance the effects of targeted therapy treatments (10). Most of the immune checkpoint genes are upregulated in ccRCC, and thus, they indicate a tumor in an immune-hot (high immune infiltration inside the tumor) condition. Compared with immune-cold (lack of immune infiltrates) tumors, the higher levels of infiltrating lymphocytes in the nidus could help eliminate tumor cells, resulting in a better prognosis (11). By affecting the TME and proliferation of immune cells, potassium channels are involved in the tumorigenesis, proliferation, and migration of tumors (12). As reported by Masi A (13), hERG1 voltage-dependent potassium channels promote the secretion of vascular endothelial growth factor from tumor cells, especially in high-grade gliomas. This stimulates neoangiogenesis and enhances the progression of malignancy. Moreover, high expression of TREK-1, a two-pore domain potassium channel, in prostate cancer increases the proliferation of tumors, and the overexpression of Kv1.1 potassium channels promotes the proliferation of breast cancer (14, 15).

Previous studies (16, 17) have proven that potassium channels can affect the immune system. In particular, T lymphocytes need potassium channels to activate to enhance the tumor. This leads to the avoidance of immune destruction or the promotion of inflammation, which is associated with cancer progression and prognosis. However, the effect of potassium channels on the intratumoral immune microenvironment of ccRCC remains to be investigated. Thus, this study was designed to evaluate the correlation between potassium channels and the TME of ccRCC.

## Materials and methods

### Public data acquisition and processing

In this study, transcriptome RNA sequencing (RNA-seq) data of human ccRCC samples were downloaded *via* The Cancer Genome Atlas (TCGA) (https://portal.gdc.cancer.gov/). All the RNA-seq data selected in our study were normalized by fragments per kilobase million (FPKM). After removing duplications and samples that were missing data, the KIRC data set consisted of 29 normal samples and 394 cancer samples and matched the clinical information of the selected data. The RNA-seq data were combined into an expression profile matrix by Perl (http://www.perl.org/). The “org.hs.eg.db” package was used to convert the Ensembl ID into a gene symbol. Our study used GeneCards (https://www.genecards.org/) to obtain PCRGs.

### Human renal clinical tissues and RNA extraction

ccRCC tumor tissues and adjacent normal tissues were collected from 12 patients who underwent radical nephrectomy at The First Affiliated Hospital of Jinan University, and RNA was extracted from those tissues. These patients had WHO/ISUP grades I to IV. This study was approved by the Ethics Committee of the First Affiliated Hospital of Jinan University. Both patients and the control individuals provided written informed consent.

The total RNA of tumor tissues and adjacent normal tissues from all patients was extracted using the EZ-Press RNA Purification Kit (EZbioscience, USA). cDNA was obtained by reverse transcription using the PrimeScript RT Kit (TaKaRa, Japan).

### Identification of prognostic differentially expressed PCRGs

The “limma” package was used to identify the differentially expressed genes (DEGs) between ccRCC tumor and adjacent normal tissues. Genes with an adjusted P< 0.05 and |log2 fold change (FC)|>0 were defined as DEGs. Additionally, the “survival” package was used to perform univariate Cox regression, and the screening condition was P< 0.05 to identify prognostic genes. Based on the above results, the PCRGs obtained from GeneCards (https://www.genecards.org/) were used to screen differentially expressed PCRGs and prognostic PCRGs. The intersection of the two was used to identify prognostic differentially expressed PCRGs. To explore the correlations and interactions among these genes, the “igraph” package was used to draw a correlation graph of the prognostic differentially expressed PCRGs. The protein–protein interaction (PPI) network of these genes was constructed and clustered through STRING (https://string-db.org/).

### Construction and evaluation of the PCRG signature

The TCGA-KIRC cohort was divided into a training cohort (n=275) and a validation cohort (n=117). Due to the large number of PCRGs, our study used least absolute shrinkage and selection operator (LASSO) regression to identify PCRGs that significantly impacted survival in the training set and calculated their regression coefficients. The PCRG signature was used to calculate the risk score of each patient, and the PCRG expression value of each patient was multiplied by the corresponding coefficient of the gene for weighting. Then, the weighted expression values of the 10 PCRGs were added to finally obtain the risk score of the patient, which was calculated as follows:

Risk score=


$$\sum_{i=1}^{n}Exp_{i}*Coef_{i}$$

where n is the number of genes in the PCRG signature, i.e., n=10, *Exp*_*i*_  the expression value of the ith gene of the patient, and *Coef*_*i*_ is the coefficient of the gene in the PCRG signature.

The patients were classified into high-risk and low-risk groups according to the median risk score, and then time-dependent receiver operating characteristic (ROC) analysis was used to verify the prediction accuracy of the signature. Multivariate Cox regression was used to verify whether the risk score obtained by the signature was an independent prognostic factor, and Kaplan–Meier survival analysis was performed to detect whether there was a significant difference in survival between the high- and low-risk groups. A heatmap was used to show the expression of the 10 PCRGs that constituted this signature in ccRCC. Principal component analysis (PCA) was used for dimension reduction, and the expression pattern of PCRGs in high- and low-risk patients was studied. Furthermore, one-way ANOVA was used to analyze whether the risk scores of grade, stage, T stage, and M stage at different levels were different.

### Construction and evaluation of the nomogram

A nomogram was constructed based on sex, age, stage, T stage, M stage, and the risk score to predict ccRCC patient overall survival (OS) at 1, 3, and 5 years. The concordance index (C-index), calibration curve and decision curve analysis (DCA) were used to evaluate the predictive accuracy of the nomogram.

### Functional enrichment analysis and gene set enrichment analysis

After classifying the samples of the TCGA-KIRC cohort into high-risk and low-risk groups according to the median risk score, the “limma” package was used to search for DEGs. The screening conditions were P< 0.05 and |log2FC|>0. These DEGs were used for Gene Ontology (GO) and Kyoto Encyclopedia of Genes and Genomes (KEGG) functional enrichment analyses. In addition, gene set enrichment analysis (GSEA) was used to uncover which biological functions the DEGs showed statistically significant and consistent differences in.

### Estimation of the TME

The “Cibersort” package was used to analyze the abundance ratios of 22 types of immune cells in the TCGA ccRCC cohort and determine whether the PCRG signature could distinguish different immune cell infiltrations. The “survival” and “survminer” packages were used to analyze the relationships between immune activators and the PCRG signature and the effect of the expression of immune activators on the survival of patients in the high- and low-risk groups.

### Gene mutation analysis

The “maftools” package was used to analyze the tumor mutation burden (TMB) based on somatic mutation data from TCGA. We calculated the TMB for each patient and compared the TMB between the high-risk and low-risk groups.

### Prediction of sensitivity to chemotherapy

Based on the Genomics of Drug Sensitivity in Cancer (GDSC) database, we used the “pRRophetic” package to calculate the half-maximal inhibitory concentration (IC50) for different chemotherapy drugs between the high-risk and low-risk groups.

### Real−time quantitative PCR

Based on the SYBR Green (ChamQ Universal SYBR qPCR Master Mix, Vazyme Biotech, China)method, the CFX96 real-time PCR system (Bio-Rad, USA) was used for RT–qPCR detection. After the expression level of GAPDH was used for normalization, the relative expression level of mRNA was determined. The mRNA-specific primer sequences are shown in **Table 1**.

**Table 1:** mRNA-specific primer sequences.

Gene	Primer sequence	Tm
*ATP1A3*	F: GCAGTGTTTCAGGCTAACCAGG	58.9
	R: CTCCTTCACGGAACCACAGCA	60.2
*GNB3*	F: CGTTTGGCCCTGTGACTAT	55.0
	R: TACCAGGGTGCTACACTTTA	52.3
*GNB4*	F: TCCTATCCAAAGGCATCCACA	54.0
	R: TGTTCAGTTGACCACGAGTGT	56.0
*NSF*	F: GTGTCACATTGCCCCTCTG	56.6
	R: TCTGGTCTATTGGTCATTCCTG	53.7
*GAPDH*	F: ACAGTTGCCATGTAGACC	54
	R: TTTTTGGTTGAGCACAGG	52

### Statistical analysis

Statistical analyses were conducted using R 4.1.1 and GraphPad Prism 8 (GraphPad Software, Inc.). All data are expressed as the mean ± SD. A paired difference test between ccRCC samples and adjacent normal samples in the two groups by the “limma” package was used to determine the DEGs. Analysis with one-way ANOVA followed by the Student–Newman–Keuls multiple comparison test was used for the comparison of three or more experimental groups. For qPCR data, Student’s t test was used for analysis.

## Results

### Identification of differentially expressed prognostic PCRGs in the TCGA ccRCC cohort

**Figure 1:**
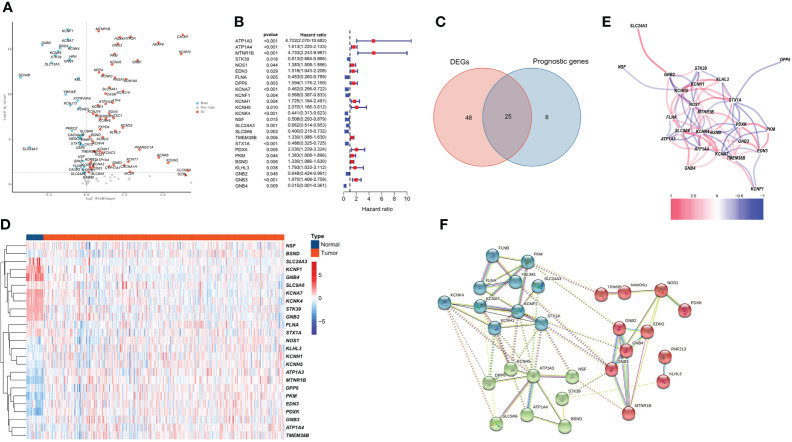
Identification of 25 prognosis-associated differentially expressed PCRGs. **(A)** Volcano plot of DEGs between ccRCC and normal tissues. **(B)** Univariate Cox analysis of 25 differentially expressed PCRGs in ccRCC. **(C)** Venn diagram showing the intersection of the DEGs and prognostic genes. **(D)** Heatmap illustrating the differential expression of 25 prognosis-associated PCRGs between ccRCC tissues and normal tissues. **(E)** Correlation between 25 differentially expressed PCRGs in the TCGA-KIRC cohort. Red represents a positive correlation, and blue represents a negative correlation. **(F)** PPI network of 25 PCRGs.

Among 118 PCRGs, 73 were differentially expressed. Of these, 44 were upregulated, and 29 were downregulated in tumor tissues (**Figure 1A**). Seventy-three prognosis-related PCRGs were obtained by univariate Cox regression, and the screening threshold was *p*< 0.05 (**Figure 1B**). The intersection of differentially expressed PCRGs and prognosis-related PCRGs was used to obtain 25 differentially expressed PCRGs (**Figure 1C**). The heatmap illustrates the different expression patterns of these PCRGs in ccRCC and normal tissues (**Figure 1D**). We examined the correlation between 25 differentially expressed PCRGs in the TCGA-KIRC cohort. Red dots represent a positive correlation, and blue dots represent a negative correlation (**Figure 1E**). Our study mapped the correlations among the 25 PCRGs and constructed the PPI network of these genes through the STRING database. The results showed that the 25 PCRGs could form 3 clusters (**Figure 1F**).

### Construction and validation of the PCRG signature

**Figure 2:**
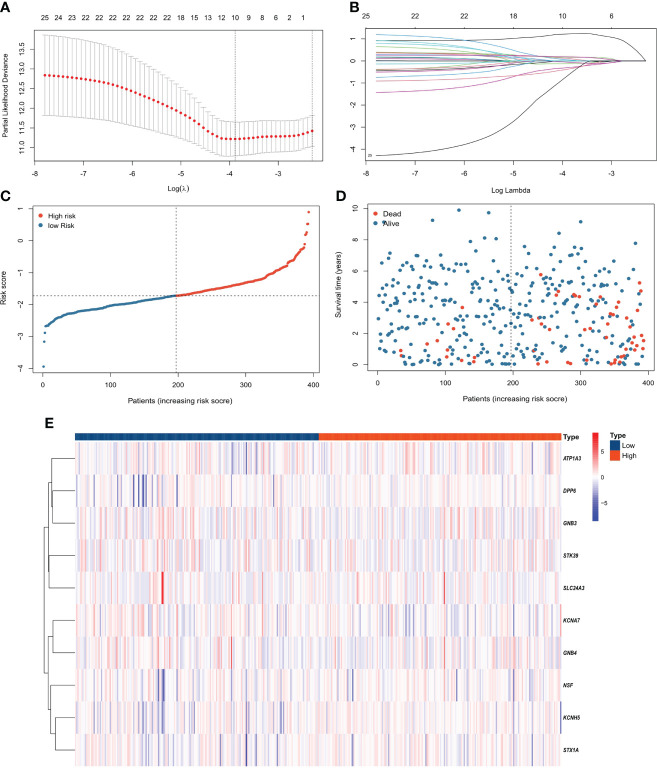
Construction of the PCRG prognostic signature. **(A)** Selection of the optimal parameter (λ) of LASSO regression through cross-validation. **(B)** LASSO coefficient profiles of the 10 genes that comprise the prognostic signature selected by λ. **(C)** The TCGA-KIRC cohort was divided into high-risk and low-risk groups according to the median risk score value. **(D)** Higher mortality was observed in the high-risk group than in the low-risk group. **(E)** Heatmap of the expression levels of 10 PCRGs in the high-risk and low-risk groups.

Compared with λ_1SE_, λ_min_ has higher accuracy. Hence, λ_min_ was selected to build the model for accuracy in our study. The LASSO algorithm was used to determine Log(λ_min_) = -3.8 (**Figure 2A**), and the PCRG prognostic signature consisting of 10 genes (**Figure 2B**) was established. The specific gene composition and coefficient of each gene are shown in **Table 2**. The PCRG prognostic signature was used to calculate the patients’ risk scores and divide them into high-risk and low-risk groups (**Figure 2C**). The risk score calculated by the signature can separate surviving patients from nonsurviving patients (**Figure 2D**). In addition, the heatmap shows the expression patterns of the 10 genes that make up the PCRG prognostic signature between the high-risk and low-risk groups (**Figure 2E**).

**Table 2:** Genes and their coefficients that constitute the PCRG signature.

Gene	Coefficient
***ATP1A3***	1.20856795
***GNB3***	0.192896088
*SLC24A3*	0.165657175
*DPP6*	0.141493127
*STK39*	0.087752983
*STX1A*	-0.0357362
*KCNA7*	-0.126888902
*KCNH5*	-0.33762536
***NSF***	-0.437328756
***GNB4***	-0.977379864

Genes in bold font we performed qPCR validation, and the remaining genes were not.

**Figure 3:**
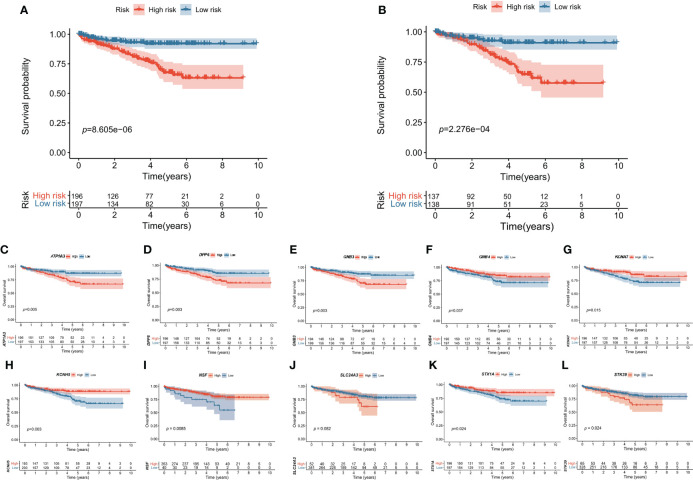
Kaplan–Meier survival curves of the high-risk and low-risk groups. The overall survival of the high-risk group was significantly lower than that of the low-risk group in the **(A)** training cohort and **(B)** validation cohort. The effect of each gene **(C–L)** expression value on OS in the prognostic signature.

**Figure 4:**
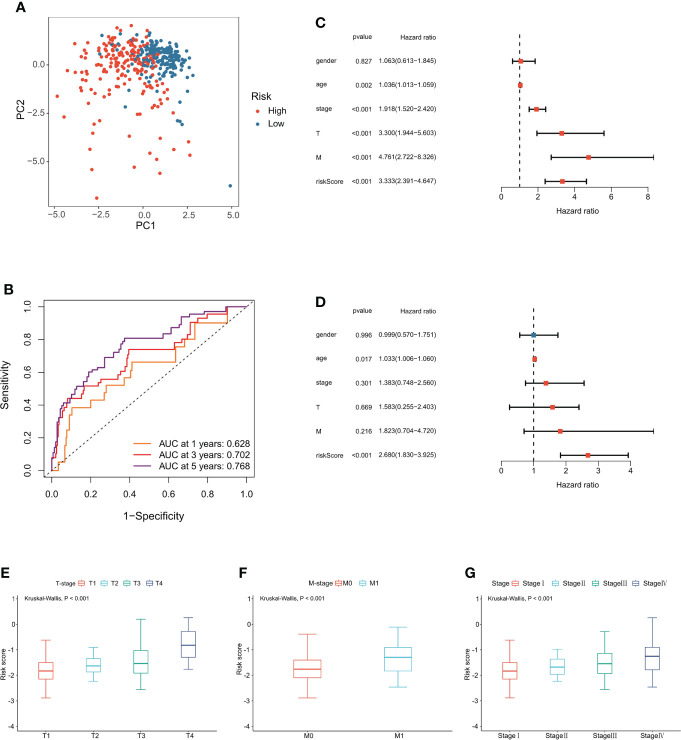
Evaluation of the PCRG prognostic signature. **(A)** The low-risk and high-risk groups can be separated into two parts using PCA. **(B)** Time-dependent ROC curves for the risk score for predicting 1-, 3-, and 5-year survival in the TCGA-KIRC cohort. **(C)** Univariate Cox and **(D)** multivariate Cox regression analyses of age, sex, grade, stage, T stage, M stage, and risk score. Relationship between the risk score and **(E)** T stage, **(F)** M stage, and **(G)** stage.

The signature was significantly correlated with survival in the training cohort (**Figure 3A**) and validation cohort (**Figure 3B**). Nine of the 10 genes that constituted the prognostic signature were significantly associated with the Kaplan−Meier survival curve (**Figures 3C–L**). PCA showed that the risk score could categorize patients with different risk scores into two groups (**Figure 4A**). ROC curve analysis was used to illustrate the accuracy of this signature. The 1-year, 3-year, and 5-year area under the curve (AUC) values of the risk score were 0.628, 0.702, and 0.768, respectively. Interestingly, the 1-year, 3-year, and 5-year AUC values increased gradually, suggesting that the PCRG signature has an excellent ability to predict long-term prognosis (**Figure 4B**). The forest map shows that the hazard ratio (HR) of the risk score and 95% confidence interval (CI) were 3.333 (2.391−4.647), *p*<0.001, in univariate Cox regression (**Figure 4C**) and 2.680 (1.830−3.925), *p*<0.001, in multivariate Cox regression (**Figure 4D**). Moreover, with the increase in T stage (**Figure 4E**), M stage (**Figure 4F**), and stage (**Figure 4G**), the risk score also increased. These findings suggest that the higher the malignancy degree of ccRCC was, the higher the risk score.

### Construction and evaluation of the nomogram

**Figure 5:**
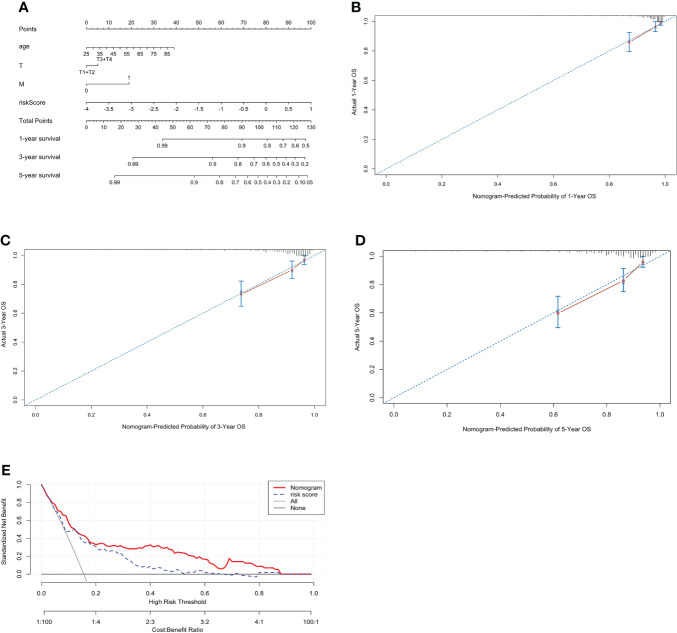
Construction of a prognostic nomogram including the risk score for ccRCC. **(A)** A nomogram for predicting the 1-, 3- and 5-year overall survival of individual ccRCC patients. The calibration curve for predicting the 1-year **(B)**, 3-year **(C)**, and 5-year **(D)** overall survival of ccRCC patients. The better the red line and the 45° dashed line fit, the better uniformity between the nomogram-predicted and actual probabilities. **(E)** DCA curves of the nomogram and risk score.

To further evaluate the predictive ability of the PCRG signature, we constructed a prognostic nomogram for ccRCC based on the different weights of the risk score, stage, T stage, M stage, sex, and age (**Figure 5A**). Our study evaluated the consistency between nomogram-predicted survival and actual survival using the C-index, and the C-index of the nomogram was 0.76. The calibration curves (**Figures 5B–D**) of the nomogram showed that the OS predicted by the nomogram was in good agreement with the actual OS. The DCA curves indicated that the nomogram provided a better net benefit (**Figure 5E**).

### Functional annotation analysis of the PCRG signature

**Figure 6:**
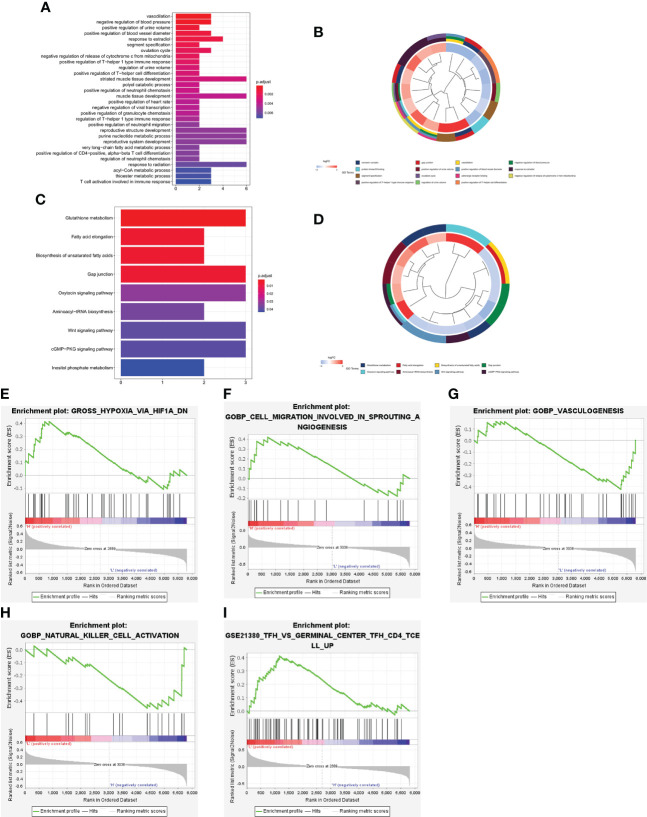
Functional enrichment analysis of the DEGs between the high-risk and low-risk groups. Bar plot **(A)** and circle plot **(B)** of the top 30 GO pathway analysis enrichment results. Bar plot **(C)** and circle plot **(D)** of KEGG pathway analysis enrichment results. **(E–I)** GSEA between the high-risk and low-risk groups.

To further explore the underlying biological mechanisms involved in the association between the PCRG signature and ccRCC, GO and KEGG were used to annotate the 84 DEGs between the high-risk and low-risk groups. According to GO enrichment analysis (**Figures 6A, B**), the DEGs are mainly involved in the “positive regulation of T-helper 1 type immune response”, “positive regulation of T−helper cell differentiation”, “positive regulation of neutrophil migration”, “positive regulation of CD4 -, Alpha-beta T-cell differentiation”, “T-cell activation involved in the immune response” and other immune-related pathways. The KEGG pathways (**Figures 6C, D**) were mainly related to metabolism, gap junctions, tumor-related signaling pathways, and other biological processes closely related to tumorigenesis and development. In addition, GSEA of the high-risk and low-risk groups showed that the high-risk group was positively correlated with hypoxia (NES=1.67, FDR=0.04), angiogenesis (NES=1.65, FDR=0.04), and vasculogenesis (NES=1.93, FDR=0). In contrast, the low-risk group was positively correlated with NK-cell activation (NES=-1.84, FDR=0.03) and germinal center formation (NES=1.72, FDR=0.04) (**Figures 6E–I**).

### Association between immune cell infiltration and TMB and the risk score in ccRCC

**Figure 7:**
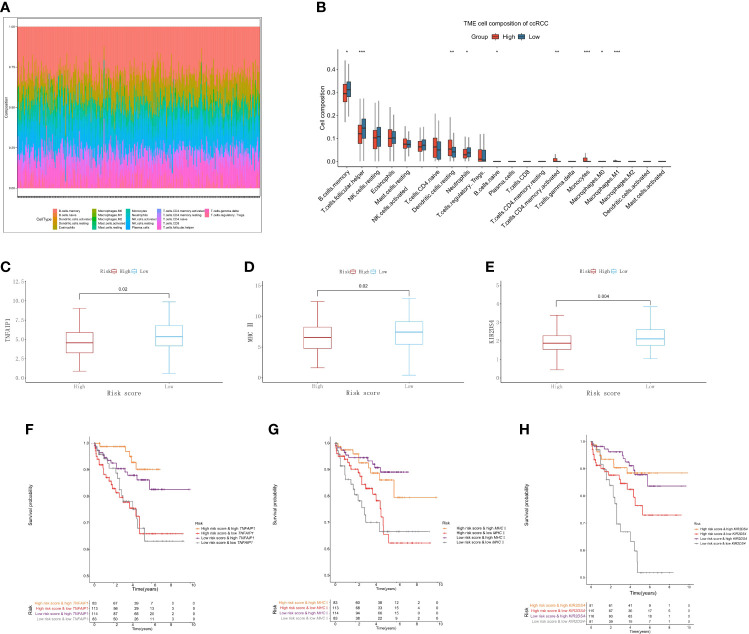
Immune cell infiltration associated with the risk score in ccRCC. **(A)** The abundance ratios of 22 immune cells in the TCGA-KIRC cohort. **(B)** Differences in immune cell abundance between the high-risk and low-risk groups. Relationship between the risk score and the immune activators TNFAIP1 **(C)**, MHC II **(D)**, and KIR2DS4 **(E)**. Relationship between the risk score and the expression of the immune activators TNFAIP1 **(F)**, MHC II **(G)**, and KIR2DS4 **(H)** with OS.

To further verify the results of functional enrichment analysis and GSEA, the present study compared the infiltration of immune cells in the high- and low-risk groups (**Figure 7A**). Most of the immune cells were more infiltrated in the low-risk group than in the high-risk group, especially memory B cells, NK cells and T helper cells, as mentioned in the above results (**Figure 7B**). These findings suggest that the risk score may be related to the formation of tertiary lymphatic structures (TLSs) in ccRCC. In addition, our study explored the relationship between the risk score and the immune activators TNFAIP1, MHC II and KIR2DS4. The results showed that the lower the risk score was, the higher the expression of these immune activators (**Figures 7C–E**). After combining these results with the PCRG signature, the prognosis of the high-risk + low immune activator group was significantly worse than that of the low-risk + high immune activator group (**Figures 7F–H**).

### Association between TMB and the risk score in ccRCC

**Figure 8:**
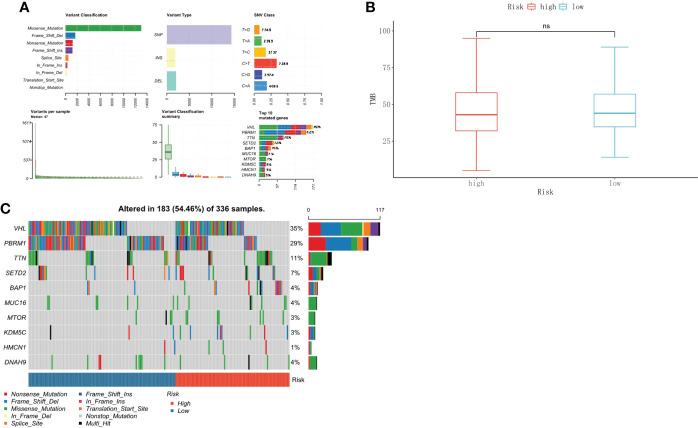
Tumor mutational burden associated with the risk score in ccRCC. **(A)** The overall landscape of somatic mutations. **(B)** TMB comparison between the high-risk and low-risk groups. **(C)** Waterfall maps of the somatic mutations in the high-risk and low-risk groups.

We further analyzed the relationship between TMB and the risk score in ccRCC. The somatic mutation results showed that most genomic variants were missense mutations. The rest were frameshift deletion mutations, nonsense mutations, and frameshift insertion mutations, and C>T was the most common SNV type in both the high- and low-risk groups (**Figure 8A**). From an overall perspective, the samples in the low-risk group had a significantly larger number of variants than those in the high-risk groups (**Figure 8B**). The top 10 most frequently mutated genes in the corresponding groups are illustrated in **Figure 8C**. *VHL*, *PBRM1*, and *TTN* occupied the top three positions in both groups.

### Prediction of chemotherapeutic drug responses

**Figure 9:**
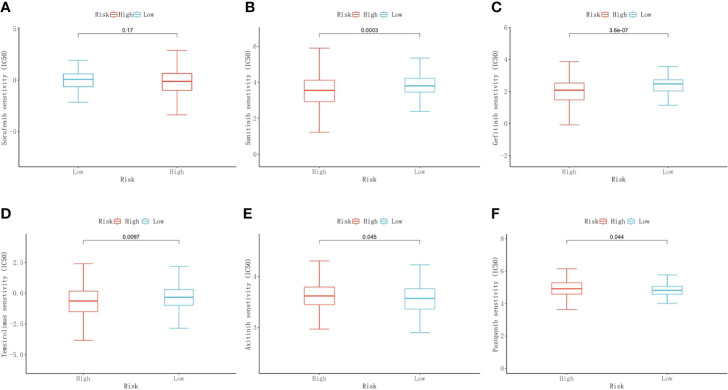
Predictive results of chemotherapeutic responses. The differences in the chemotherapeutic response to common chemotherapeutic drugs between the high- and low-risk groups **(A–F)**.

We used the “pRRophetic” package to predict the chemotherapeutic response to commonly used chemotherapy agents in the high- and low-risk groups based on drug sensitivity data from GDSC. The results showed that there was no difference in response between the two groups for sorafenib. The low-risk group demonstrated a higher response to sunitinib (*p*<0.001), gefitinib (*p*<0.001), and temsirolimus (*p*=0.0097) than the high-risk group. The response to axitinib (*p*=0.045) and pazopanib (*p*=0.044) was higher in the high-risk group than in the low-risk group (**Figures 9A–F**).

### The expression of key genes in the PCRG signature in ccRCC

**Figure 10:**
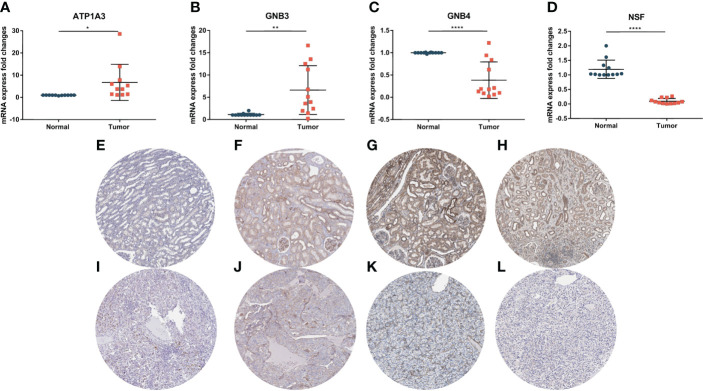
Expression of key genes in the PCRG prognostic signature in ccRCC and normal kidney tissues. The mRNA expression levels of ATP1A3 **(A)**, GNB3 **(B)**, GNB4 **(C)**, and NSF **(D)** in clinical samples were detected by qPCR. Immunohistochemistry of ATP1A3, GNB3, GNB4, and NSF in normal tissues **(E–H)** and ccRCC tissues **(I–L)** from the Human Protein Atlas (HPA) database.

To verify the authenticity of the PCRG signature, we collected tumor and normal tissues from 12 ccRCC patients in this study. RNA was extracted for RT–qPCR to verify the PCRG signature. The gene with the most significant coefficient made the most decisive contribution to the risk score. *ATP1A3* and *GNB3* had the largest positive coefficients in the signature, and *GNB4* and *NSF* had the largest negative coefficients. Therefore, *ATP1A3*, *GNB3*, *GNB4*, and *NSF* were identified as key genes in the signature and further analyzed. The expression of *ATP1A3* (**Figure 10A**) and *GNB3* (**Figure 10B**) in tumor tissues was significantly higher than that in normal tissues.

In comparison, the expression of *GNB4* (**Figure 10C**) and *NSF* (**Figure 10D**) in tumor tissues was significantly lower than that in normal tissues, suggesting that these key genes play an essential role in the occurrence and development of ccRCC. The results of RT–qPCR confirmed the database analysis conclusion. In addition, we used the Human Protein Atlas (HPA) online database (https://www.proteinatlas.org/) to detect the protein expression of key genes. The immunohistochemical results of *ATP1A3*, *GNB4*, and *NSF* were consistent with the RT–qPCR results (**Figures 10E–L**).

## Conclusions

With the rapid development of high-throughput sequencing technologies, we can better understand the cancer biology of ccRCC. In this study, we constructed a novel prognostic signature composed of PCRGs. The PCRG signature could accurately classify patients in the training and validation cohorts into high- and low-risk groups. Our results demonstrate that the PCRG signature has high specificity and sensitivity and can supplement clinicopathological parameters for prognosis evaluation and treatment guidance for patients. We analyzed the TME landscapes of the high- and low-risk groups. The results showed that the low-risk group had higher proportions of immune cell infiltration and somatic mutations and a better response to chemotherapy. These findings suggest that patients in the low-risk group were more likely to benefit from immunotherapy and chemotherapy, indicating that the PCRG signature has better performance than other prognostic signatures. In addition, by combining the PCRG signature with clinical parameters such as age, T stage, and M stage, we constructed a nomogram to provide clinicians with a robust and straightforward method for the personalized evaluation of ccRCC patients. Finally, we found that the mRNA expression of the four key genes in the PCRG signature in clinical samples was consistent with their coefficients.

## Discussion

In this study, we established a prognostic signature consisting of potassium channel-related genes (PCRGs) to predict the prognosis of patients with clear cell renal cell carcinoma (ccRCC) by bioinformatics methods. The risk score calculated by the PCRG signature was strongly associated with the prognosis of patients with ccRCC, especially for long-term prediction. In short, the PCRG signature we propose here may be a complementary method for assessing the prognosis of patients with ccRCC.

As a fatal malignant tumor, ccRCC is a common pathological type of renal cell carcinoma (RCC) that accounts for approximately 80% of all RCCs. Due to its high degree of drug resistance and 20-40% recurrence rate after surgical resection, the prognosis of these patients is poor, and the quality of human life is seriously affected (18–20). Therefore, it is of great significance to find new biomarkers or targets for the early diagnosis and intervention of ccRCC. It has been reported that potassium channels are involved in the proliferation and migration of ccRCC. For example, overexpression of the potassium inward rectifier channel *KCNJ1* can inhibit the proliferation and migration of ccRCC and lead to apoptosis. Its low expression is related to the poor prognosis of ccRCC (21). Another study reported that the Ca^2+^-activated potassium channel KCa3.1 is highly expressed in ccRCC and promotes ccRCC metastasis, which is associated with worse survival (22). Previous studies have shown that potassium channels, such as voltage-gated Kv1.3 and the Ca2^+^-activated potassium channel *IKCa1*, are crucial for the activation and proliferation of T lymphocytes (23, 24) and can be used as drug targets to regulate the function of the immune system (25). According to this research, Kv1.3 is highly expressed in the perivenular and parenchymal inflammatory infiltrates of multiple sclerosis (MS) brain tissue on T cells from the cerebrospinal fluid (26). Moreover, the use of Kv1.3 inhibitors can specifically and permanently block the proliferation and function of CD4+ T cells (27, 28). Furthermore, the activation of Kv1.3 on T lymphocytes can enhance the NLRP3 inflammasome and increase the secretion of IL-1β, which strengthens the T-cell-mediated inflammatory response (29).

Recently, the tumor microenvironment (TME), which includes tumor-infiltrating immune cells (TICs), was shown to play a decisive role at all stages of tumor progression. The high level of immune infiltration of ccRCC has been proven in many types of studies. Therefore, potassium channels are likely to affect the tumor and immune system, which could affect the modeling the TME. Ultimately, this could lead to the occurrence and development of ccRCC. How potassium channels directly lead to cancer remains unclear, and only a few studies have been carried out on the correlation between PCRGs and the development of ccRCC (21, 22).

Our study first proposed a prognostic signature consisting of 10 PCRGs that could predict the prognosis of patients with ccRCC, especially for long-term prediction. The low-risk group calculated by the PCRG signature had a better prognosis and overall survival (OS) than the high-risk group. We analyzed the differentially expressed genes (DEGs) between the high-risk and low-risk groups predicted by the PCRG signature through GO enrichment analysis. The results showed that those genes were mainly concentrated in T lymphocyte activation and regulation, which is consistent with the previously reported literature that suggest that potassium channels could regulate T lymphocytes. Additionally, KEGG pathway analysis showed that the DEGs were mainly related to tumor-related signaling pathways and tumorigenesis. This result also supports the participation of potassium channels in the development of ccRCC. GSEA showed that the low-risk group was positively correlated with follicular helper CD4 T cells (TFHs) and germinal centers (GCs).

In contrast, the high-risk group was positively related to hypoxia, angiogenesis, vasculogenesis, and glycolysis. In addition, we compared the infiltration of immune cells in ccRCC tumor tissues and normal tissues. We found more infiltration of immune cells, especially memory B cells, NK cells, and T helper cells, in normal tissues than in ccRCC tissues. These results suggest that tertiary lymphoid structure (TLS) formation may be underway.

TLS is a lymphocyte aggregate located in nonlymphoid tissue (30). TLSs do not exist under physiological conditions but occur as the result of infection, autoimmunity, chronic inflammation, and even numerous cancers (30). They exhibit all the characteristics of structures in the lymph nodes associated with the generation of an adaptive immune response, including a T-cell zone with mature dendritic cells (DC), a germinal center with follicular DCs, proliferating B cells, and high endothelial venules (HEV) (31). Previous studies have identified TLSs as a tumor prognostic biomarker and therapeutic target that is associated with improved survival (30, 32, 33). Our results show that the numbers of TFH, GC, CD4+ T cells, and memory B cells predicted by the PCRG signature were higher in the low-risk group than in the high-risk group. These findings indicate a better prognosis and higher OS in the low-risk group. This indicates that PCRGs may affect TLS formation, including GC, by regulating T lymphocytes, such as TFH, and ultimately affect the occurrence and development of ccRCC.

Mutations in the genome of tumor cells may produce new antigens with immunogenicity that can be recognized by T lymphocytes (34). Tumor mutation burden (TMB) can reflect the tumor gene mutation status. In short, the higher the TMB is, the more tumor gene mutations are present. Thus, the possibility of forming an immunogenic new antigen is greater, and the possibility of patients benefiting from tumor immunotherapy is greater (35). Therefore, we conducted a TMB prediction analysis on the high- and low-risk groups. The mean TMB scores of the low-risk group were higher than those of the high-risk group. These findings suggest that the low-risk group may be more likely to benefit from tumor immunotherapy and to have a better response to targeted drugs and chemotherapeutic drugs. This was proven by our prediction of chemotherapeutic drug response to ccRCC between the high- and low-risk groups by using the PCRG signature.

Related studies have reported that PCRGs play an important role in the development of multiple diseases. For example, the G protein beta3 subunit (*GNB3*) could be a candidate gene in disorders associated with poor immune response. It has been reported that the counts of CD4+ T cells with the *GNB3* homozygous 825T allele (TT) genotype were significantly enhanced compared to those with the C825 allele (CC) genotype (36). Na^+^/K^+^‐ATPase is widespread in eukaryotic cell membranes, and its different α/β isoforms (*ATP1A1‐1A4*, *ATP1B1‐1B3*) were identified in humans in their early years (37). Moreover, the high expression of sodium pumps was shown to be closely related to the occurrence, development, and malignancy of cancer (37). Recently, *ATP1A3* has been reported to exert significant effects in various cancers, including glioblastomas (38), hepatomas (39), and medulloblastomas (40). It has been reported that bufalin inhibits the growth of hepatocellular carcinoma (HCC) cells, which is correlated with the expression level of *ATP1A3* in HCC cells (39). Another study reported that activation of *ATP1A3* could sensitize glioblastoma cells to temozolomide (41). However, the role of PCRGs in the development of ccRCC has not been reported, and further research is needed. In this study, through a series of rigorous analyses, we established a prognostic signature consisting of PCRGs that could predict the prognosis of patients with ccRCC. Our results suggest that these key genes may play a significant role in the occurrence and development of ccRCC. The PCRG signature may improve our understanding of the role of potassium channels in the occurrence and development of ccRCC and provide a reference for discovering new prognostic biomarkers and immunotherapy methods for ccRCC.

There were some limitations to our study. First, the robustness of the prognostic signature needs to be verified by external data sets. However, there is no suitable ccRCC gene expression data set, so we have to split the TCGA-KIRC cohort into training and validation cohorts to partially compensate for the study’s limitations. Second, our results require further basic experiments and clinical studies to validate and further explore the potential underlying mechanisms and clinical applications of PCRGs in ccRCC. Finally, many factors, such as comorbidities, influence overall survival, but we did not study them in depth. Therefore, further studies concentrating on RFS/CSS are required.

## Data availability statement

The original contributions presented in the study are included in the article/supplementary material. Further inquiries can be directed to the corresponding authors.

## Ethics statement

This study was approved by the Ethics Committee of the First Affiliated Hospital of Jinan University. Both patients and controls provided written informed consent.

## Author contributions

Conception and design of the research: All authors. Acquisition of data: RZ and YL. Analysis and interpretation of data: RZ, YL, and MS. Statistical analysis: RZ and YL. Drafting manuscript: RZ, YL, DH, MS, YW, and TC. Obtaining funding: JH, JG, WH, JC, and YZ. All authors contributed to the article and approved the submitted version.

## Acknowledgments

Financial support from the Science and Technology Program of Guangzhou, China (805147677069) is gratefully acknowledged. The experimental instrument support from the Medical Experimental Center, School of Medicine, Jinan University is gratefully acknowledged.

## Conflict of interest

The authors declare that the research was conducted in the absence of any commercial or financial relationships that could be construed as a potential conflict of interest.

## Publisher’s note

All claims expressed in this article are solely those of the authors and do not necessarily represent those of their affiliated organizations, or those of the publisher, the editors and the reviewers. Any product that may be evaluated in this article, or claim that may be made by its manufacturer, is not guaranteed or endorsed by the publisher.
